# Dietary perception and knowledge among cardiac patients visiting tertiary care hospital outpatient department (OPD)

**DOI:** 10.12669/pjms.40.6.8289

**Published:** 2024-07

**Authors:** Maryam Zahid, Eman Jamil Khan, Asif Nadeem, Hafsa Khalil

**Affiliations:** 1 Maryam Zahid, MS, MSc (Food and Nutrition). Armed Forces Institute of Cardiology and National Institute of Heart Disease, Rawalpindi, Pakistan; 2 Eman Jamil Khan, MBBS. Frontier Medical & Dental College, Abbottabad, Pakistan; 3 Asif Nadeem, FCPS (Cardiology). Armed Forces Institute of Cardiology and National Institute of Heart Disease, Rawalpindi, Pakistan; 4 Hafsa Khalil, MS. Armed Forces Institute of Cardiology and National Institute of Heart Disease, Rawalpindi, Pakistan

**Keywords:** Cardiac, Dietary, Knowledge, Tertiary care hospital

## Abstract

**Objective:**

To appraise the dietary perception and knowledge of cardiac patients visiting Outpatient department of a tertiary care hospital.

**Methods:**

A cross sectional study of 466 patients attending cardiac outdoor clinic at a tertiary care cardiac hospital in Rawalpindi, Pakistan were selected through convenient sampling from January to April 2021. Patients included in the study were above 18 years of age and had a cardiac disease. A structured questionnaire in English and Urdu was drafted with questions related to patient demographics, dietary perception and knowledge.

**Results:**

Among 466 patients, 60% (n=280) were males and 40% (n=186) were females whereas 14% (n=66) of the patients were uneducated and 15% (n=68) had completed postgraduate education. In this study 23% (n=106) of the participants used internet to search nutrition related information and 72 % (n=339) of the patients consider their lifestyle to be healthy. Majority (n=261, 56%) of the patients had no idea regarding sodium consumption. 46% (n=215) of the patients had no knowledge regarding the effects of bakery items on cardiac health.

**Conclusion:**

Dietary Knowledge plays a predominant role in the management of cardiac disease. The study concluded that cardiac patients had inadequate knowledge regarding dietary intake in cardiac disease and has high prevalence of dietary myths and misconceptions. Strategic plan including nutritional awareness program and intensive counseling sessions should be designed to increase dietary knowledge of cardiac patients.

## INTRODUCTION

Non-communicable diseases are responsible for major cause of death, disability, and disease, especially cardiovascular diseases (CVD), including coronary artery disease, arrhythmias and other vascular diseases.^1^ It is estimated that genetics are only half of the reasons that can trigger CVD. In other words, the other half of the reasons are expected to be acquired factors, including unhealthy diet.^2^,^3^ It is seen even genetic predisposition of CVD can be reversed by following a healthy lifestyle.^4^ It is important to acknowledge that acquired factors, including diet, are often associated with CVD.

In Asia centers a survey was carried out which reported that only less than two thirds of the participant have received the services of a clinical dietitian. The survey further stated that lack of time and educational material are barriers in receiving nutritional education.^5^ Furthermore there are numerous variability and unreliability in the dietary information provided by different healthcare workers to patients. Information from unconstrained sources such as the internet/television, unbidden advice from relatives/friends and socio-cultural beliefs present locally further confuse the people.^6^

In order to start a successful nutritional education program it is important to know the knowledge, attitude and practices affecting eating patterns of the community, this motivated us to carry out this study. The objective of the study was to understand the dietary perception and knowledge of cardiac patients visiting OPD of a tertiary care hospital. This will help in developing the nutrition education plan for patients.

## METHODS

We conducted a prospective cross sectional questionnaire-based study of 466 patients attending cardiac Outdoor clinic at a tertiary care cardiac hospital in Rawalpindi, Pakistan from January 2021 to April 2021. Patients were selected through convenient sampling. All the patients, consenting to participate were recruited to fill the questionnaires and were enrolled after their implied consent. Patients above 18 years of age, had a known cardiac disease and were able to give informed consent were included. Patients with comorbidities were excluded from the study.

### Ethical Approval:

The Hospital Ethics Committee and Institutional Review Board approved the study. (Letter No. 29/8/R&D/2019/13). The participation was entirely voluntary. Informed verbal consent was taken from all the participants before inquiring regarding their knowledge.

A structured objective questionnaire in English and Urdu was modified^7^ after a methodical review of literature about the eating pattern and dietary choices of cardiac patients. The questionnaire was divided into two sections. The first section contained questions relating to patient demographics (age, gender, ethnicity, education level, ethnicity of the patient). In the second section; questions related to dietary perception and knowledge were included. This section contained 19 questions relating to dietary beliefs and food related behavior in cardiac and hypertensive patients (Table-I). The Cronbach alpha coefficient for the 19 questions was 0.65, suggesting that the items have relatively moderate internal consistency. Sample size was estimated to be 323 using open epi sample size calculator with 95% confidence interval and prevalence of 57%.^8^ However, for the better understanding of the population’s dietary perception sample size was increased to 466.

**Table-I:** Descriptive statistics including demographics.

Demographics (N= 466)	Data Mean ± SD / % (frequency)
Age in yrs. (mean)	51.5±15.6
Male	60% (280)
Female	40% (186)
***Ethnicity***	
Punjabi	60% (280)
Pukhtoon	15% (70)
Kashmiri	8% (36)
Others	10% (49)
Patients reluctant to disclose ethnicity	7% (33)
***Education***	
Uneducated	14% (66)
Uncompleted primary education	16% (75)
Completed primary education	14% (66)
Completed secondary education	22% (104)
Completed bachelor’s degree	19% (87)
Completed postgraduate education	15% (68)

Statistical analysis was performed using SPSS version 24 by considering 95% confidence interval and 5% margin of error, P-value: less than 0.05 was considered significant. Descriptive statistics was applied to calculate mean ± SD of continuous variables e.g. age and for categorical variables like gender, ethnicity etc. frequency (percentage) was calculated. To compare the variables, pearson’s chi-square test and independent sample T-test was applied.

## RESULTS

A total of 466 study participants were included in this study. The mean age of the study participants was 51.5±15.6 years. 280 (60%) were male and 186 (40%) were females. Among 466 patients, only 41% (n=183) watch nutrition related television programs. In our population 76% (n=354) don’t use internet to search nutrition related information. Demographic detail of the study population is shown in Table-I.

Out of 183 patients who watch nutrition related television program 61% (n: 111) believe restricting all kinds of fat is required to be healthy (P-value: 0.002). 36% (n=65) believe low carb diets are good choices for weight loss (P-value: 0.001). 21% (n=38) believe there is no difference between bran and brown bread (P-value: 0.002). Out of 106 patients who use internet to search nutrition related information; 55% (n=58) think bakery items like biscuits are healthy snacks for cardiac patients (P-value: <0.0001). About 21% (n=22) patients believe that no Sodium means to avoid salt only (P-value: <0.001). Other responses of the patients are mentioned in Table-II.

**Table-II:** Responses of the patients regarding dietary perception and knowledge.

1	You consider their lifestyle is healthy	72%(N:339)	26%(N:119	2% (N:8)	-
2	You believe in hot and cold food theory?	71%(N:330)	19%(N:88)	10%(N:47)	0.2%(N:1)
3	Avoid egg because of cardiac disease?	45%(N:211)	45%(N:210)	9%(N:42)	1%(N:3)
4	Refined flour products are fattening?	51%(N:239)	31%(N:143)	17%(N:81)	1%(N:3)
5	Restrict all fats to be healthy?	67%(N:311)	26%(N:120)	7%(N:34)	0.2%(N:1)
6	Is Olive oil best when cooked?	86%(N:401)	5%(N:23)	9%(N:41)	0.2%(N:1)
7	Low carb diets are good choices for weight loss?	29%(N:134)	22%(N:100)	48%(N:224)	2%(N:8)
8	Brown rice are those white rice which are cooked till they are brown?	17%(N:330)	33%(N:155)	49%(N:228)	1.1%(N:5)
9	Parched grams are good choice for cardiac patients?	69%(N:323)	18%(N:83)	12%(N:57)	1%(N:3)
10	No sodium means to avoid salt only?	20%(N:96)	20%(N:95)	56%(N:261)	1%(N:3)
11	Bakery items are heart healthy snacks?	38%(N:179)	15%(N:70)	46%(N:215)	1%(N:4)
12	There is no difference between bran and brown bread?	17%(N:79)	37%(N:170)	46%(N:213)	1%(N:4)
13	Fried fish is healthy for heart?	72%(N:333)	25%(N:114)	4%(N:19)	
14	Organic egg is more nutritious than white egg?	75%(N:349)	18%(N:83)	8%(N:31)	
15	Drinking fruit juices is as healthy as fruit?	34%(N:159)	59%(N:277)	6%(N:26)	1%(N:4)
16	Packed fruit juices are healthy options?	16%(N:76)	79%(N:369)	4%(N:20)	0.2%(N:1)
17	Nutritional content of chicken and mutton is same?	8%(N:36)	87%(N:406)	5%(N:23)	0.2%(N:1)
18	Coconut oil is better than canola oil?	19%(N:89)	45%(N:208)	36%(N:168)	0.2%(N:1)
19	Tea whitener can be used instead of milk?	5%(N:21)	89%(N:415)	6%(N:28)	0.4%(N:2)

Majority of the study participants who were well educated (post-graduated; 60.3%) use internet to gain dietary knowledge in their routine (p=<0.001), 41.8% follow ketone rich diet (p=<0.001), 69.1% use refined flour in their daily routine (p=0.005) and 66.2% considered digestive biscuits to be healthy (p=0.159). Mostly participants believed in hot and cold theory (p=0.025), try to restrict fatty food (p=<0.001), knows olive oil is healthy (p=0.386), prefer organic eggs (p=0.907), knew that the consumption of whole fruit is healthier than fruit juice (p=0.188) and intake of packed juice (p=0.032) is not healthy for cardiac patients. Very few of the study population had knowledge regarding the consumption of sodium i.e. they think that sodium is only present in salt (p=<0.001), likewise study population considered fried fish (p=0.089) to be healthy. Mostly did not know that consumption of brown rice (0.446), bran bread (p=0.101), mutton instead of chicken (p=0.255) and coconut oil as compared to canola oil (p=0.068) are considered to be nutritious.

## DISCUSSION

The management of cardiovascular disease not only needs the medical intervention and pharmacological regimen but also intensive education on heart healthy diet. This study reports the dietary perception and knowledge of cardiac patients which will further help to design a nutritional education program for awareness of cardiac patients.

In our study majority (n=261, 56%) of the patient visiting hospital outdoor clinic have no idea regarding the sodium consumption. According to Khan ,^9^ the perception of healthiness tends to reduce when we increase sodium content or additives, fats, and carbohydrates in our diet. In this study half of the respondents gave inaccurate for the question **“**hypertension dietary was only restricted for salt**”**.

Our (n=401, 86%) majority of study participants think the best way to consume olive oil is through cooking, which is an incorrect perception as phenolic content and antioxidant capacity of the olive oil reduce after cooking. Santosa et al.^10^ found that consumers in United States of America have hardly any knowledge of the effects of olive oil on health, but olive oil is contemplated to be an authentic and natural product.

In our study approximately half (n=211, 45%) of the population avoids egg because of the cardiac disease, which is a good choice as they are reducing cholesterol intake and facilitating long-term survival and health.^11^Eggs are considered an excellent source protein with multiple vitamins and mineral. It also has high quantity of dietary cholesterol (350 mg/100 g), it elevates serum total, LDL- and HDL-cholesterol levels. These all are major risk factors of CVD, although they are considered clinically insignificant.^12^

In this study majority (n=333, 72% ) of the patients believe that fried fish is healthy which is an imprecise information as frying the fish not only decreases its nutritional content but also make it an unhealthy choice. According to Rafique et al.,^13^educated population have awareness regarding the benefit of fish in diet and can afford it which increases its consumption, whereas it is observed that the risk of cardiovascular event is escalated if more than two servings per week of fried fish is consumed.^14^

Majority (n=208, 45%) of our participants perceive coconut oil is not a better option as compare to canola oil in cardiac patient. It’s a right perception of our cardiac patient as coconut fat has 80% saturated fat is not recommend, it increases low-density lipoprotein.^15^According to Lima and Block recently coconut oil has allured the attention of worldwide population. The benefits of using coconut oil regularly include cholesterol-lowering effect, healthy weight loss, reduction of the risk of cardiovascular diseases (CVDs) and others.^16^

Our majority (79%, n=369) population believed that drinking packed juice is an healthy option and 59% (n=277) of the people believed that drinking juices is a better option as compared to eating fresh fruit whereas Consuming diet which is rich in legumes, vegetables, wheat, chicken and fruits is protective against the risk of premature acute myocardial infarction in a Pakistani population.^17^

In our study majority (n=415, 89%) of the participants think tea whitener can’t be used regularly. It is right approach of the cardiac patients of not preferring tea whitener over milk in their daily routine as it is bad for cardiac health.^18^ Non-dairy tea whiteners or tea whiteners are a substitute to milk which have an ability to whiten the tea. Multiple researches have proved that tea whiteners effect our health adversely as it increases 69%of the cholesterol, 98% increase in triglyceride, and 84% increase in LDL levels.^19^

### Significance of study:

Annually millions of people die due to inadequate food habits resulting in various diseases. Lack of knowledge may be one of the reasons of inadequate food habit. . The patient’s misconceptions or knowledge gained from dubious sources of information may be the cause of their ignorance. Evaluating one’s understanding of nutrition can help combat false information and encourage healthy eating habits. These findings will not only play an important role in determination of the nutritional knowledge of the cardiac patients but it will have meaningful impact in the development of Strategic plan to increase dietary knowledge of cardiac patients. There is an alarming need for social campaigns that promote healthy eating habits. Numerous US initiatives promoting healthy lifestyles have shown extremely encouraging outcomes in recent years.^20^

### Limitations:

Our study is limited as convenience sampling technique was chosen in which patients were recruited at only one cardiac tertiary care hospital. Our results may not be generalizable to patients in other settings.

## CONCLUSION

Dietary Knowledge and perception play an important role in the management of hypertension and cardiac disease. The study concluded that cardiac patients had inadequate knowledge regarding dietary intake in cardiac disease and has high prevalence of dietary myths and misconceptions. Strategic plan should be designed to increase dietary knowledge of cardiac patients. Being brief and empirical, it would be useful to use this tool in other settings to assess the knowledge level of patients on coronary artery diseases. Research findings will help clinicians and public health workers to develop educational programs about the management of cardiac risk factors though diet and nutrition.
